# The Medical Work of the Local Government Board

**Published:** 1893-11-11

**Authors:** 


### The Medical Work of the Local Government Board.

### IV.—LOCALITY AND DISEASE.

We sometimes meet with newspaper paragraphs telling of mysterious epidemics in various parts of the country—diseases such as never were before, coming and raging, and disappearing with the spontaneity and wilfulness of the wind which bloweth where it listeth. The average reporter is not a pathologist, and he loves to make a sensation; hence arises the mystery he talks about. When a scientist is put on the track, it vanishes like the mystery of a crime before a well-trained detective. Two such "mysterious outbreaks" are narrated in the report of the Local Government Board.

In Raunds, a shoemaking village in Northamptonshire, such an epidemic took place. It was generally defined as pneumonia, but was frequently accompanied by the symptoms of meningitis, and was also marked by sore throat. Young children were usually the first to suffer, and the pneumonia attacked none over twenty-five, though tonsillitis alone was found in all ages. The disease differed from ordinary pneumonia in being infectious, though sometimes the difficulty of tracing the contagion gave it an air of mystery. It appears, too, that it could be communicated from one person to another through an intermediary who escaped the disease. For example, a schoolgirl, whose home was two miles out of the village, regularly spent her dinner-hour at a house in Raunds, where a baby was suffering from a typical and severe attack of the epidemic. She herself escaped, but the baby at her home, who had never been brought to the village, nearly died of the so-called pneumonia, and her father and mother were troubled with sore throat at the same time. The mother of the girl went out to work at a neighbouring farmhouse. There the two youngest children fell ill with all the characteristic symptoms of the prevailing complaint, an elder boy took a sore throat, the father had tonsillitis, and the grandmother was laid down with an attack of what was, rightly or wrongly, called pleurisy. Thus, in an isolated community, there occurred an apparently spontaneous attack of the epidemic then raging in Raunds, and the only explanation of its appearance that can be offered is that it was carried from the village by this girl, who herself did not suffer from it. The medical men of Raunds regarded the disease as infectious, and held that the average period of incubation was four days, though in mild cases the whole attack occupied only five or six days. The average duration, however, was ten to twelve days. While the pneumonia was the prominent feature in every case, the accompanying symptoms varied considerably. Sometimes there was delirium, screaming, convulsions, coma, retraction of the head, and similar tokens of meningitis; other patients suffered from a rash which their mothers thought was chicken-pox, while the doctors diagnosed it as sudamina, and in some cases, where desquamation followed, surmised the presence of scarlet fever. The ailment altogether was of a complicated character, which defied codification under any one disease, although in all its variations the pneumonic type was predominant.

Raunds is an industrial village, with cottages built rather closely together; Heyford, twenty-two miles distant, is an agricultural village, with ample space between the houses. It has the advantage over Raunds of a gravel subsoil, and of fairly good sanitary arrangements. Yet in the last week of February, 1891, when the epidemic of pneumonia was dying away in Raunds, a similar epidemic arose and ran its course in Heyford. The only difference in the two instances was that whereas at Raunds the disease was more markedly pneumonic, it was the meningitis that showed itself most clearly at Heyford. As in Raunds, children were the chief sufferers, and when the disease entered a house it generally attacked more than one member of the family. Whence came this mysterious epidemic? It would not be wise to speak with any assurance, but it deserves to be noted that towards the end of the summer of 1890 a family of gipsies, encamped on the roadside near Raunds, suffered from an attack of pneumonia similar to that which subsequently appeared in the village. They went away, but the disease remained, and considering their nomadic habits, it seems to be not unlikely that they visited and infected Heyford too.

Another instance of the mysterious springing up of an epidemic occurred at Bush Hill Park, between Enfield and Edmonton. In this case it was scarlatina that appeared, seemingly without any inducing cause. But on inquiry it was found that all the families suffering from the disease obtained their milk from one dairyman. This man resented the suggestion that his milk conveyed the infection, and, indeed, the three cows he kept himself were free from any symptom of disease. But it transpired that these cows supplied only the "nursery" milk required by his customers, and that the larger quantity of his supply came from a farm in Essex. To this milk the infection was promptly traced. In the milkman's own family the baby, fed on the milk of the home cows, was well and thriving, while an elder child who had the Essex milk was attacked at the same time as his customers, and this distinction was marked in other families which received the double supply. The inquiry was then transferred to Essex. The farmer who supplied the Bush Hill dairyman sent him nearly all his milk, retaining only some for the use of his family, and a small quantity which he sold to a shopkeeper in the village, and the children in both families suffered from scarlatina at the same time as the epidemic occurred at Bush Hill. The villagers in general could not afford to buy new milk, which in this instance was rather fortunate for them than otherwise, but nevertheless betrays a sad condition of affairs among our peasantry. Our agricultural labourers are indeed poor when, though employed on a dairy farm, they cannot afford to buy milk.

But it is evident that there is such a demand for milk in London that purchasers are not too particular as to its quality, for the Essex farmer lost only two days' milk through the epidemic at Bush Hill. On the third he entered into a contract to supply a dairy in the East-end of Londen, and this with the concurrence of the local medical officer of health, who would not see any connection between the milk and the Bush Hill scarlatina. Nevertheless, there seems no doubt that it was the source of infection. Some ill-luck in his cows calving had made the farmer send away his stock and have his cowsheds thoroughly cleansed and disinfected. He then restocked his farm by degrees, and among the cows he bought were three who were suffering from "boils" on the udder. The milk from all the cows was mixed before being sent away, and any infection that might come from the milk of the diseased cows was thus at once diminished and diffused. The scarlatina at Bush Hill was the result. This inference may seem too hasty; but, at least, Dr. Klein who took specimens of the diseased udders, and made cultures of them in various media, found in these numerous specimens of the same microbe which characterised the well-known outbreak of scarlet fever at Hendon. And when the Bush Hill dairyman ceased to get his milk from the Essex farm, no new cases of fever occurred among his customers. The Local Government Board, by tracing epidemics to their source, proves them to be preventable, and thus leads the way to their ultimate prevention.

